# Practical Approach to the Diagnosis of the Vulvo-Vaginal Stromal Tumors: An Overview

**DOI:** 10.3390/diagnostics12020357

**Published:** 2022-01-31

**Authors:** Giuseppe Angelico, Stefano Marletta, Giuseppe Broggi, Paolo Vigneri, Giada Maria Vecchio, Lucia Salvatorelli, Gaetano Magro

**Affiliations:** 1 Pathology Unit, Cannizzaro Hospital, 95126 Catania, Italy; giuangel86@hotmail.it; 2 Department of Diagnostics and Public Health, Section of Pathology, University and Hospital Trust of Verona, 37134 Verona, Italy; stefano.marletta@gmail.com; 3 Department of Medical, Surgical Sciences and Advanced Technologies “G.F. Ingrassia”, Anatomic Pathology, University of Catania, 95123 Catania, Italy; giuseppe.broggi@gmail.com (G.B.); giadamariavecchio@gmail.com (G.M.V.); lucia.salvatorelli@unict.it (L.S.); 4 Department of Clinical and Experimental Medicine, University of Catania, 95123 Catania, Italy; vigneri.p@unict.it

**Keywords:** deep angiomyxoma, cellular angiofibroma, angiomyofibroblastoma, myofibroblastoma, vulvovaginal region, review

## Abstract

Background: The category of the “stromal tumors of the lower female genital tract” encompasses a wide spectrum of lesions with variable heterogeneity, which can be nosologically classified on the basis of their morphologic and immunohistochemical profiles as deep (aggressive) angiomyxoma (DAM), cellular angiofibroma (CAF), angiomyofibroblastoma (AMFB) or myofibroblastoma (MFB). Despite the differential diagnosis between these entities being usually straightforward, their increasingly recognized unusual morphological variants, along with the overlapping morphological and immunohistochemical features among these tumours, may raise serious differential diagnostic problems. Methods and Results: The data presented in the present paper have been retrieved from the entire published literature on the PubMed website about DAM, CAF, AFMB and MFB from 1984 to 2021. The selected articles are mainly represented by small-series, and, more rarely, single-case reports with unusual clinicopathologic features. The present review focuses on the diagnostic clues of the stromal tumours of the lower female genital tract to achieve a correct classification. The main clinicopathologic features of each single entity, emphasizing their differential diagnostic clues, are discussed and summarized in tables. Representative illustrations, including the unusual morphological variants, of each single tumour are also provided. Conclusion: Awareness by pathologists of the wide morphological and immunohistochemical spectrum exhibited by these tumours is crucial to achieve correct diagnoses and to avoid confusion with reactive conditions or other benign or malignant entities.

## 1. Introduction

The category of the “stromal tumours of the lower female genital tract” covers a wide spectrum of lesions with variable morphological and immunohistochemical heterogeneity. They arise from the specialized, hormonally responsive stroma of the lower female genital tract, and, based on morphological and immunohistochemical features, at least four tumour entities can be nosologically recognized: (i) deep (aggressive) angiomyxoma (DAM); (ii) cellular angiofibroma (CAF); (iii) angiomyofibroblastoma (AMFB) and (iv) myofibroblastoma (MFB) [1,2]. Among these tumours, it is crucial to distinguish DAM from the others due to its relatively high risk of local recurrence. Differential diagnosis between these entities is usually straightforward if the typical morphology and clinicopathologic features are encountered. However, some tumours may share several morphological and immunohistochemical features, along with unusual morphologies, raising serious differential diagnostic problems. As MFB, CAF and AMFB may also share chromosomal aberrations, namely a 13q14 deletion (MFB and CAF) or *MTG1–CYP2E1* fusion transcripts (AMFB and MFB), it is likely that they are histogenetically related, as previously suggested [3,4,5]. Notably, a recent article emphasized the possibility that CAF may occur with other mesenchymal tumours showing the same 13q14 deletion, such as spindle-cell lipoma and mammary-type MFB [6].

Based on these morphological, immunohistochemical and cytogenetic findings, the hypothesis that vulvovaginal CAF, MFB and AMFB are in the spectrum of a single entity, likely arising from a common precursor stromal cell of the lower female genital tract, has been postulated [3,4,5,6,7]. The present overview focuses on the diagnostic clues of the stromal tumours of the lower female genital tract to aid in achieving correct classification. The main clinicopathologic features of each single entity, emphasizing their differential diagnostic clues, are discussed and summarized in tables. Representative illustrations, including their unusual morphological variants, of each single tumour are also provided. Awareness by pathologists of the wide morphological and immunohistochemical spectrum exhibited by these tumours is crucial to achieve a correct diagnosis and to avoid confusion with reactive conditions or other benign or malignant entities.

## 2. Materials and Methods

The data presented in the present paper have been retrieved by the entire published literature on the PubMed website about DAM, CAF, AFMB and MFB from 1984 to 2021. The selected articles were mainly represented by small-series (due to the relative rarity of these tumours) and, more rarely, by single-case reports with unusual clinicopathologic features. The histological illustrations have been retrieved from a personal consultation series of DAM, CAF, AMFB and MFB (63 cases) by Prof. G. Magro. The morphological and immunohistochemical diagnostic clues for each single tumour are provided. In addition, the unusual morphological features that can be diagnostically challenging are emphasized in the form of tables.

## 3. Results

### 3.1. Deep (Aggressive) Angiomyxoma (DAM)

**Figure 1:**
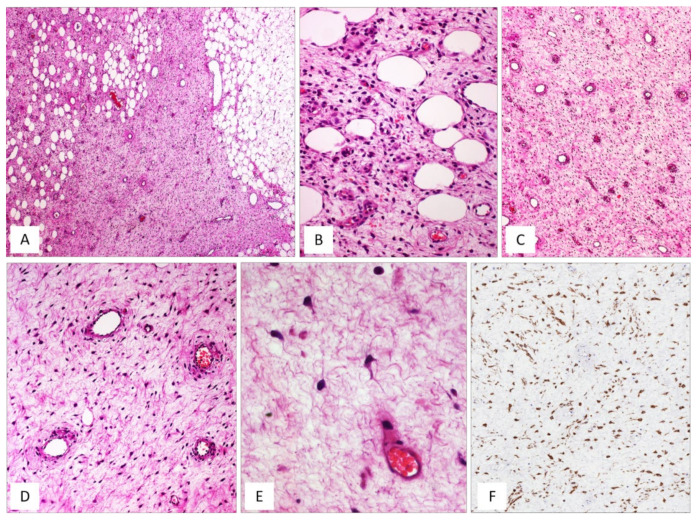
DAM, classic type. (**A**) Tumour showing infiltrative margins into the surrounding adipose tissue (H and E, original magnification 50×). (**B**) Higher magnification: showing neoplastic cells intermingling with adipocytes (H and E, original magnification 200×). (**C**) Low magnification: showing a myxoid hypocellular tumour with numerous interspersed thin-walled blood vessels (H and E, original magnification 50×). (**D**) Tumour is composed of small-sized spindled or stellate cells (H and E, original magnification 200×). (**E**) Higher magnification: neoplastic cells exhibiting dendritic cytoplasmic processes (H and E, original magnification 300×). (**F**) Diffuse and strong immunoreactivity for desmin supports the myofibroblastic nature of the neoplastic cells (immunoperoxidase, original magnification 150×).

**Figure 2:**
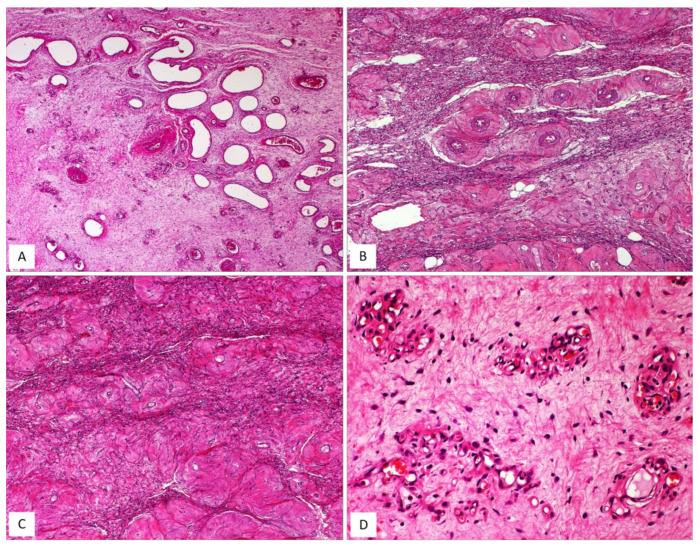
DAM. (**A**) Classic-type DAM showing variable-sized blood vessels (H and E, original magnification 50×). (**B**–**D**) Unusual vascular features in DAM: (**B**) hyalinization of the vascular walls (H and E, original magnification 50×); (**C**) hyalinization of the vascular walls with total obliteration of their lumens (H and E, original magnification 50×); (**D**) capillary-like microvascular proliferation, as seen in glioblastoma (H and E, original magnification 200×).

**Figure 3:**
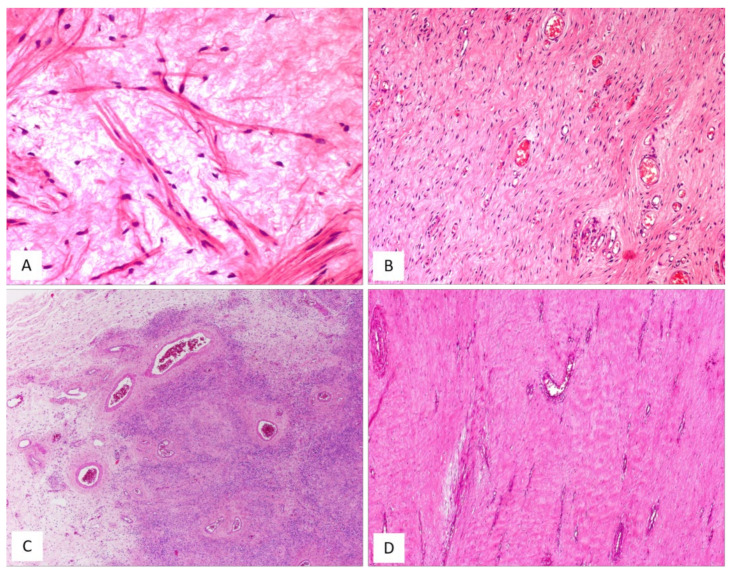
Unusual features in DAM. (**A**) Thin-sized mature smooth-muscle cells are haphazardly interspersed within myxoid tumour stroma (H and E, original magnification 300×). (**B**–**D**) Unusual features in DAM: (**B**) area with neurofibroma-like appearance; spindle cells with wavy nuclei, set in a collagenized stroma (H and E, original magnification 50×); (**C**) hypercellularity is seen around the blood vessels (H and E, original magnification 50×); (**D**) locally recurrent DAM; fibro-sclerotic tumour with interspersed thin-walled blood vessels (H and E, original magnification 50×).

DAM is a rare, locally infiltrative and non-metastasizing myofibroblastic stromal tumour first described by Steeper and Rosai in 1983 [8]. The tumour is diagnosed predominantly in reproductive-aged females, with peak incidence in the third decade [1,2,8,9]. The vulvovaginal region, perineum and pelvis represent the most common sites in women, while sporadic cases have also been reported in the inguinal region, spermatic cord, scrotum and pelvic cavity in adult males [9]. Similarly to the other stromal tumours of the vulvovaginal region, DAM is often confused with Bartholin gland cysts or inguinal hernias. Clinically, DAM presents as a relatively circumscribed large, slowly growing multilobular or polypoid mass with extension into the surrounding tissues. The cut surface shows a glistening, gelatinous appearance and ranges in size from a few centimetres to 20 cm. The classic-type morphology of DAM is that of an infiltrative, uniformly hypocellular tumour composed of small-sized spindled or stellate cells, haphazardly interspersed in an abundant myxoedematous stroma rich in fine collagen fibrils and containing numerous small-to-medium/large-sized blood vessels [8,9,10,11,12,13] (Figure 1 and Figure 2). The neoplastic cells exhibit a bland-looking morphology with poorly defined, scant cytoplasm and round, hyperchromatic nuclei; mitoses are absent or rare (Table 1 and Figure 1). In approximately 30% of cases, isolated or small bundles of thin, smooth-muscle cells are scattered within the myxoid stroma, occasionally close to blood vessels [8,13] (Figure 3).

Although the diagnosis of DAM is usually straightforward if the classic-type morphology is encountered, in a recent paper some clinicians reported that several unusual morphological features do exist, causing diagnostic problems, especially in recurrent tumours (Table 2).

By immunohistochemistry, DAM is typically a desmin-positive myofibroblastic tumour (Figure 1F) with variable expression of HMGA2 [14], α-smooth-muscle actin (from 27% to 95% of cases) and CD34 (from 17% to 50%) [13,15]. As some clinicians have previously observed in a previous article, it is likely that the morphological variations in cellular and stromal composition of DAM may reflect the plasticity of the neoplastic cells in adopting a myofibroblastic (vimentin+/desmin+/smooth-muscle actin+/−) or fibroblastic profile (vimentin+/desmin-/smooth-muscle actin-) in myxoid or fibrous stromal areas, respectively [13]. Despite its bland-looking morphology, DAM exhibits an infiltrative growth into the surrounding soft tissues and a risk of local recurrence. A wide local excision is difficult to achieve due to tumour-infiltrative margins, often evident at the histological examination alone. Recurrent tumours may show the same morphology of the primary lesion but hypercellular [11,12,13] or fibrosclerotic hypocellular tumours (Figure 3D) with hyalinized blood vessels (Figure 2B) and obliteration of their lumens (Figure 2C) can be seen [13]. In both hypercellular and hypocellular recurrent tumours, the identification of focal myxoid areas with the typical features of DAM is extremely helpful for a correct diagnostic interpretation [13]. In the past, DAM was considered to be a locally aggressive (destructive-type recurrence) tumour [1,2,10,11,12]. However, there is increasing evidence that this tumour has the tendency to locally recur in 9% to 50% of cases but the recurrence is of a non-invasive type in the majority of cases [9]. Accordingly, the original term “deep aggressive angiomyxoma” [8] has been changed into “deep angiomyxoma” (DAM) [9]. Recently, some clinicians have proposed the term “deep angiofibromyxoma” to emphasize that this tumour may frequently exhibit fibrous areas in both primary and recurrent lesions [13].

### 3.2. Cellular Angiofibroma (CAF)

**Figure 4:**
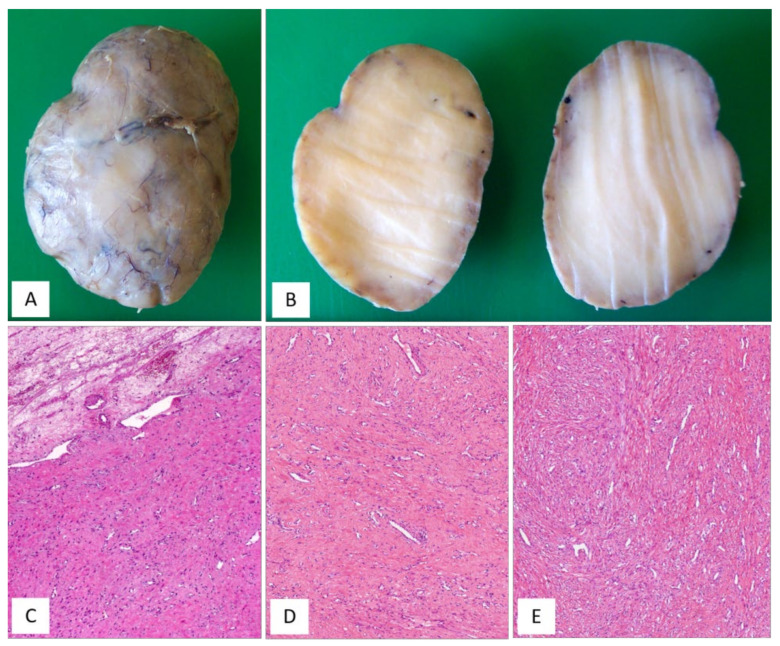
CAF, classic type. (**A**) Gross appearance: oval-shaped mass with well-circumscribed margins; (**B**) the cut surface showing a solid mass, whitish in colour. (**C**) Fibrous tumour with pushing borders (H and E, original magnification 50×). (**D**) Spindle-shaped cells set in a fibrous stroma (H and E, original magnification 50×). (**E**) Tumour area with focal fascicular arrangement (H and E, original magnification 50×).

**Figure 5:**
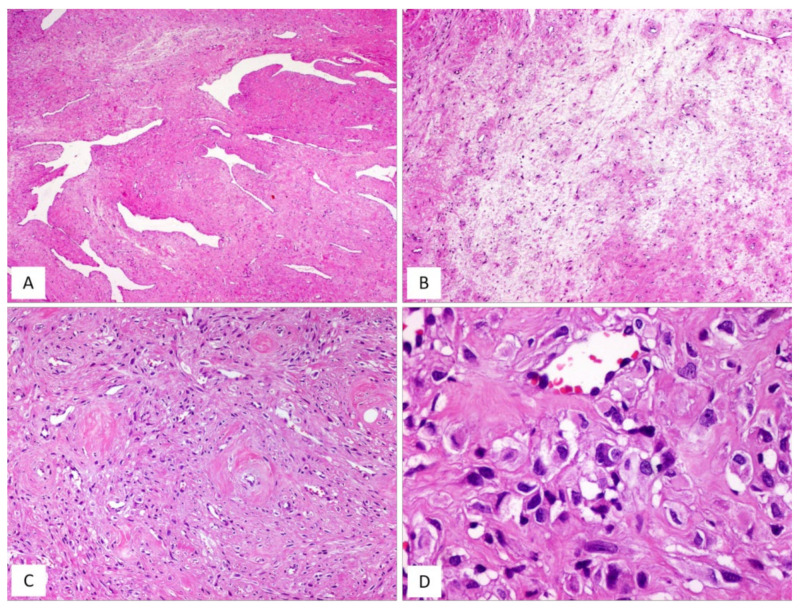
CAF: unusual features. (**A**) CAF with numerous branching, thin-walled blood vessels (H and E, original magnification 50×). (**B**) Alternating fibrous-to-mixoedematous areas (H and E, original magnification 50×). (**C**) Low magnification: showing area with atypical cells (H and E, original magnification 100×). (**D**) Higher magnification: neoplastic cells with moderate/severe nuclear atypia (so-called “*atypical CAF*”) (H and E, original magnification 300×).

CAF, originally described by Nucci et al. in 1997 [16], is a rare benign stromal tumour, fibroblastic rather than myofibroblastic in nature, usually occurring in the superficial (subcutaneous) soft tissues of the vulvovaginal region of middle-aged women [16,17]. Tumours with overlapping morphology have also been reported in the inguinoscrotal region of male patients, with the interchangeable terms “cellular angiofibroma or angiomyofibroblastoma-like tumor” [18]. Although most tumours are restricted to the pelvic area, extra-genital sites, including the retroperitoneum, pelvic and lumbar region, anus, urethra, trunk and oral mucosa have been rarely reported [17]. The most common clinical presentation is that of a slowly growing, painless mass ranging in size from 0.6 to 25 cm. Gross examination reveals a round-to-lobulated tumour mass with well-circumscribed margins. On cutting of the surface, CAF is grey-to-whitish in colour, with a firm-to-rubbery consistency (Figure 4A,B). Histologically, as its name suggests, the two main striking features of CAF are a population of spindle-shaped cells with a fibroblastic profile and a well-represented vascular component [16,17,19,20,21] (Table 3). It presents as a well-circumscribed, unencapsulated tumour, occasionally with limited infiltration of the surrounding adipose tissue [17,19]. CAF is a uniformly cellular neoplasm (moderately-to-focally highly cellular) composed of a proliferation of bland-looking spindle cells, set in a predominantly fibrous stroma containing bundles of wispy collagen fibres and numerous small- to medium-sized blood vessels, often with hyalinized walls (Figure 4C,D). The neoplastic cells, with the appearance of the fibroblasts, are cytologically bland and display oval-to-fusiform nuclei with inconspicuous nucleoli and scant, often pale-to-eosinophilic cytoplasm. They are haphazardly distributed throughout the fibrous stroma, but they may adopt a fascicular arrangement (short, intersecting fascicles) or nuclear palisading (Figure 4E). Mitotic figures are rare. In the last two decades, several papers on CAF have emphasized the possibility of unusual morphological features that can represent potential diagnostic pitfalls (Figure 5A–D and Table 4). A small subset of cases display worrisome cytologic features, ranging from severe nuclear atypia with high mitotic activity (so-called “*atypical CAF*”) to, frankly, areas of sarcomatous transformation [22,23,24]. The atypical cells can be focally dispersed within the tumour (Figure 5C,D) or, more rarely, may show a vaguely nodular configuration [22,23,24]. The cases with sarcomatous dedifferentiation show—characteristically—an abrupt transition from a CAF not otherwise specified (NOS) to a discrete sarcomatous component [21,22]. The latter can be composed of areas resembling atypical lipomatous tumour, pleomorphic sarcoma or pleomorphic spindle-cell sarcoma [22,23,24]. The immunohistochemical profile is of fibroblastic-type, being CD34-expressed in most cases [16]; variable expression of myogenic markers, including α-SMA, desmin and h-caldesmon, has been occasionally reported, in 10–20% of cases [3,17]. Nuclear immunopositivity for ER and PR and nuclear loss of RB1 is frequently observed [17]. Notably, an overexpression of p16 has been documented restricted to the atypical cells and in the sarcomatous areas [23]. Most cases of CAF show a 13q14 deletion by F.I.S.H., as shown by the monoallelic loss of *RB1* or *FOXO1* at the 13q14 locus [19,21]. CAF is a benign tumour that can rarely recur after surgical excision [19,20,25]. Notably, the cases of atypical CAF or with sarcomatous dedifferentiation (less than 15 cases reported to date) have developed neither local recurrences nor metastases [22,23,24,25].

### 3.3. Angiomyofibroblastoma (AMFB)

**Figure 6:**
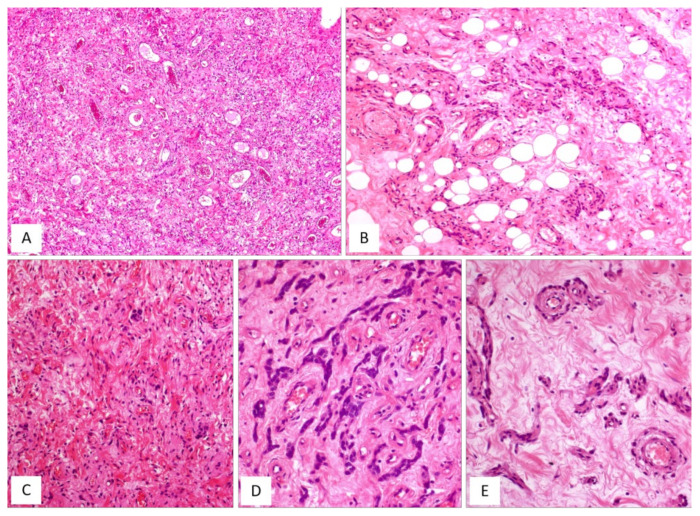
AMFB, classic-type. (**A**) Low-magnification: mesenchymal tumour with numerous thin-walled blood vessels (H and E, original magnification 50×). (**B**) Fibromyxoid stroma containing neoplastic cells, mainly arranged around the blood vessels; single adipocytes are also seen (H and E, original magnification 100×). (**C**) Higher magnification: spindle cells are haphazardly set in a myxoid stroma containing wispy collagen fibres; focally neoplastic cells with epithelioid morphology are arranged in small nests (H and E, original magnification 100×). (**D**) Neoplastic cells exhibiting a cord-like growth pattern (H and E, original magnification 200×). (**E**) The perivascular clustering of neoplastic cells is a typical feature of AMFB (H and E, original magnification 200×).

**Figure 7:**
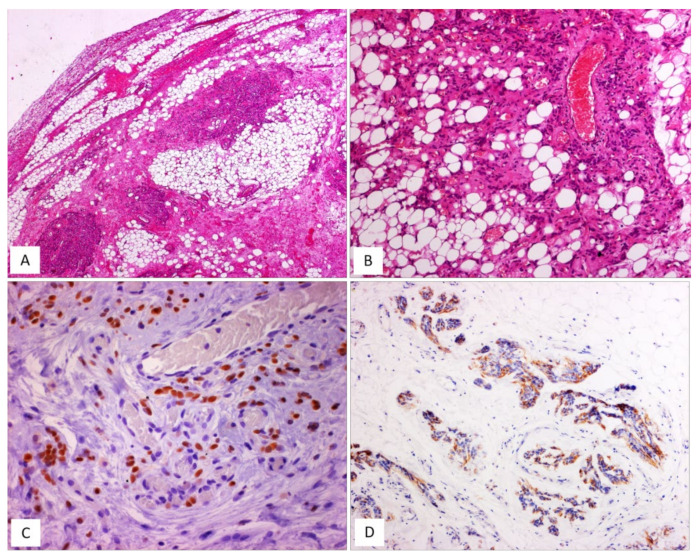
AMFB, lipomatous type. (**A**) Low-magnification: a fibrofatty tumour with well-circumscribed margins (H and E, original magnification 50×). (**B**) Neoplastic cells intermingling with mature adipocytes (H and E, original magnification 100×). Neoplastic cells are often positive for oestrogen receptors (**C**) and desmin (**D**) (immunoperoxidase, original magnification 200×, **C**, and 100×, **D**).

AMFB is a benign, superficially located (subcutaneous) stromal tumour, firstly described by Fletcher et al. in 1992 [27], that mainly involves the vulva and vagina [28,29] of women in the reproductive or, less frequently (10% of cases), in the postmenopausal years [28,29]. The less frequently affected sites include the perineum, inguinal area and fallopian tubes [27]. Tumours with partial overlapping morphology have been reported in the inguinoscrotal region of male patients under the term of “AMFB/AMFB like-tumor” [18] but they are currently best regarded as CAF [29]. Patients typically present with a slowly growing, painless subcutaneous mass/swelling, measuring <5 cm in maximum diameter, frequently misinterpreted as a Bartholin gland cyst. Grossly, AMFB presents as a well-circumscribed, usually unencapsulated lesion, typically measuring <5 cm in its greatest diameter. Rarely, AMFB may present as a large pedunculated mass [30,31]. Histologically, as its name suggests, the two main striking features of AMFB are a population of cells with a fibroblastic/myofibroblastic profile and a well-represented vascular component. Histologically, AMFB is a well-circumscribed, unencapsulated or partially/totally encapsulated tumour showing alternating hypocellular and hypercellular areas [32,33,34,35] (Table 5); it is composed of a proliferation of bland-looking spindled-to-epithelioid cells, arranged singly or in small nests or cords (Figure 6A–D) that tend to be clustered around blood vessels (Figure 6B,E). The epithelioid/plasmacytoid morphology is best appreciated in the hypercellular areas; a predominant spindle-cell morphology is more frequently observed in postmenopausal patients. The neoplastic cells, usually plump and with appreciable eosinophilic cytoplasm (better in cells with epithelioid morphology) and ovoid-to-spindle-shaped nuclei, are set in a variably myxoedematous-to-fibrous stroma (Figure 6B,C). Scattered mast cells and lymphocytes can be sparsely observed in the stroma. Mitotes are rare or absent. The vascular component is usually represented by thin-walled, capillary-like vessels (Figure 6A), but thick-walled, often hyalinized vessels, can be encountered. The presence of mature, fatty tissue, regarded as an integral part of the tumour and not merely entrapped peripheral adipose tissue, is a common feature, and, for when it represents at least 50% of the entire tumour, the term “lipomatous AMFB” has been proposed [36,37,38,39] (Figure 7A,B). Morphological variations on this common morphological theme have also been reported in AMFB (Table 6). Rarely, AMFB may contain atypical cells and high mitotic activity (so-called “malignant AMFB”) or, frankly, sarcomatous areas closely resembling leiomyosarcoma or undifferentiated pleomorphic sarcoma (so-called “dedifferentiated AMFB”) [40,41]. By means of immunohistochemistry, AMFB usually exhibits immunoreactivity for oestrogen receptors (Figure 7C), combined with a variable fibroblastic/myofibroblastic profile with the variable expression of desmin (Figure 7D) and α-smooth-muscle actin (up to 40% of cases) [5,42]. Although bcl-2, CD99, PR and AR are usually expressed in most cases, CD34 is detected, though only in a minority of cases [42]. Molecular studies have failed to detect *HMGA1* and *HMGA2* rearrangements [14] and the 13q14 deletion [36], the latter being a common finding in both CAF and MFB. In the sarcomatous component described by Nielsen et al. [40], the neoplastic cells are shown negative for desmin, SMA and CD34. Recently, the immunohistochemical strong expression of CYP2E1, as a surrogate marker of a novel genetic alteration, namely *MTG1–CYP2E1* fusion, has been reported in AMFB [5]. AMFB is a benign tumour with occasional local recurrences, especially for those tumours not completely resected. Nevertheless, the recurrences are not destructive and, thus, easy to remove. Actually, AMFB should be considered a tumour with a very low risk of sarcomatous overgrowth/dedifferentiation. Only a single case of AMFB exhibiting sarcomatous dedifferentiation has locally recurred as a purely sarcomatous tumour [40]. Distant metastases have never been reported for either “malignant or dedifferentiated AMFBs”.

### 3.4. Myofibroblastoma (MFB)

**Figure 8:**
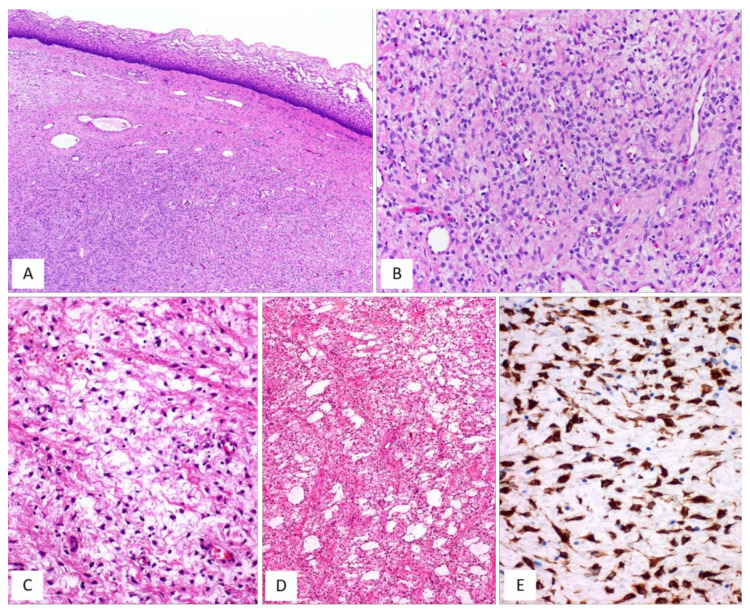
MFB, superficial type. (**A**) Low magnification: mesenchymal tumour centred in the subepithelial connective tissue; a native collagen band is seen between the tumour and the overlying squamous epithelium (so-called “*Grenz zone*”) (H and E, original magnification 25×). (**B**) Tumour is composed of small-sized spindled-to-stellate cells set in collagenous stroma (H and E, original magnification 100×). (**C**) Higher magnification: microcystic and reticular (**D**) stromal changes. Neoplastic cells are strongly and diffusely positive to desmin (**E**) ((**C**,**D**) H and E and (E) immunoperoxidase; (C) original magnifications 100×, (**D**) 50× and (**E**) 200×).

**Figure 9:**
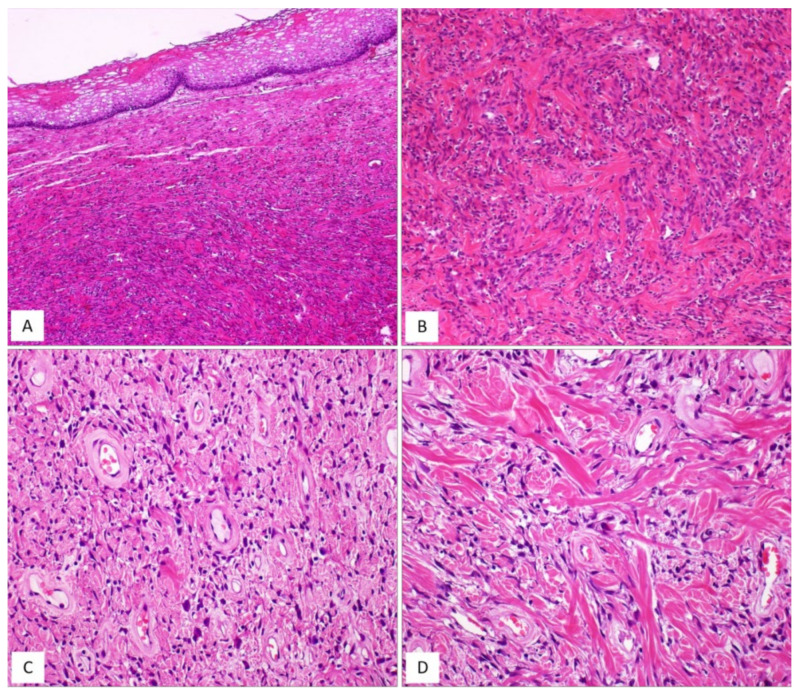
MFB, mammary type. (**A**) Low magnification: subepithelial fibrous mesenchymal tumour (H and E, original magnification 25×). (**B**) Spindle-shaped cells haphazardly arranged with interspersed thick, keloid-like collagen fibres (H and E, original magnification 50×). (**C**) Some areas may show bi- or multi-nucleated cells and hyalinized blood vessels (H and E, original magnification 50×). (**D**) Thick, keloid-like collagen fibres are a typical feature of mammary-type MFB (H and E, original magnification 50×).

MFB of the lower female genital tract is a benign neoplasm composed of spindled cells with myofibroblastic profile, that arises from the subepithelial stroma of the vagina and, less frequently, of the vulva and cervix (Table 7) [48,49,50,51,52,53,54]. MFB presents as a slowly growing, painless mass affecting mainly adults in their fifth or sixth decade of life. Grossly, tumours present as a well-circumscribed lesion of variable size (2–65 mm), showing a polypoid or nodular appearance. Histologically, MFB is a tumour centred in the subepithelial connective tissue, separated by the overlying epithelium by a thick band of native connective tissue (so-called “Grenz zone”) (Figure 8A). Two distinct subtypes of MFB can be recognized: (i) superficial MFB; (ii) mammary-type MFB. Superficial MFB is characterized by a proliferation of bland-looking, spindle-shaped or stellate cells set in a variably loose, oedematous-to-finely-collagenous stroma, often with reticular, lace-like or sieve-like changes [48,49,50,51,52,53,54] (Figure 8B–E). Tumours may exhibit a variable, moderate-to-high cellularity. The cells have a scant amount of pale-to-eosinophilic cytoplasm and oval-to-elongated-to-wavy nuclei with a small nucleolus; only rarely, mild nuclear atypia can be seen [48]. Mitotic activity is usually low (0–2 mitoses/10 high-power field). The vascular component is relatively inconspicuous and represented by small-sized blood vessels with hyalinized walls [51]. Mast cells are variably interspersed among neoplastic cells. Unlike superficial MFB, mammary-type MFB is composed of spindle-shaped cells, usually arranged in short fascicles with intervening keloid-like collagen fibres [51,52,53] (Figure 9A–D). The blood vessels often show hyalinization of their walls (Figure 9C). Immunohistochemical studies have shown that both superficial and mammary-type MFB are typically desmin+/α-SMA-myofibroblastic tumours [48,49,50,51,52,53,54] (Figure 8E). Apart from desmin, positive staining for CD99, CD34, Bcl-2, ER and PR can be detected in most cases [51]. In addition, about 90% of cases shows a loss of nuclear RB1 expression and the 13q14 deletion by F.I.S.H., confirming that MFB is pathogenetically related to other benign mesenchymal tumours showing a loss of 13q14, including spindle-cell lipoma and CAF [3]. As recently reported for AMFB, MFB also shows an overexpression of CY2E1 as a surrogate of *MTG1–CYP2E1* fusion transcripts [5]. The clinical course is benign if a complete surgical excision is achieved. Only a single patient has been reported in the literature to experience a local recurrence (after 9 years) from surgical excision [50]; however, metastatic disease has never been reported.

**Figure 10:**
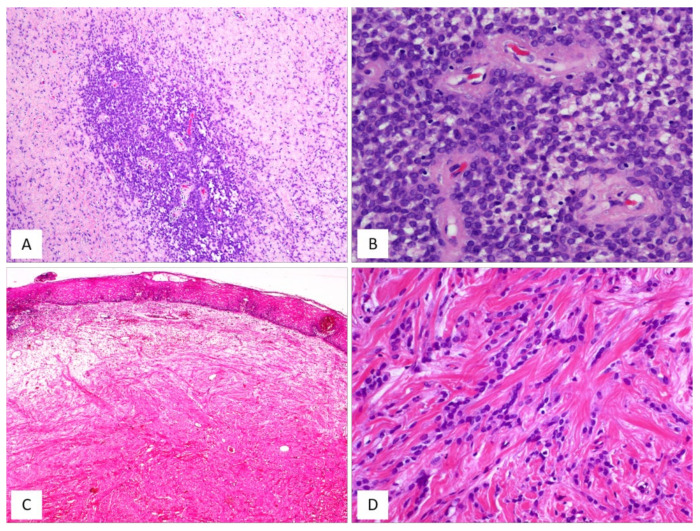
MFB, superficial/mammary type: unusual features. (**A**) Superficial-type MFB: an abrupt transition from a classic area into a hypercellular area (**B**) composed of bland-looking, small-sized, round, blue cells; mitoses and necrosis are absent (H and E, original magnifications (A) 50× and (B) 200×). (**C**) MFB, mammary type: the so-called fibrous/collagenized variant (H and E, original magnification 25×); (**D**) MFB may occasionally show, at least focally, a single-cell linear arrangement imparting to the tumour a pseudo-infiltrative growth pattern reminiscent of an invasive lobular carcinoma of the breast (H and E, original magnification 200×).

Figure 10A–D and Table 8 summarize the unusual morphologic features that may be exhibited by superficial/mammary-type MFB.

## 4. Differential Diagnosis

**Figure 11:**
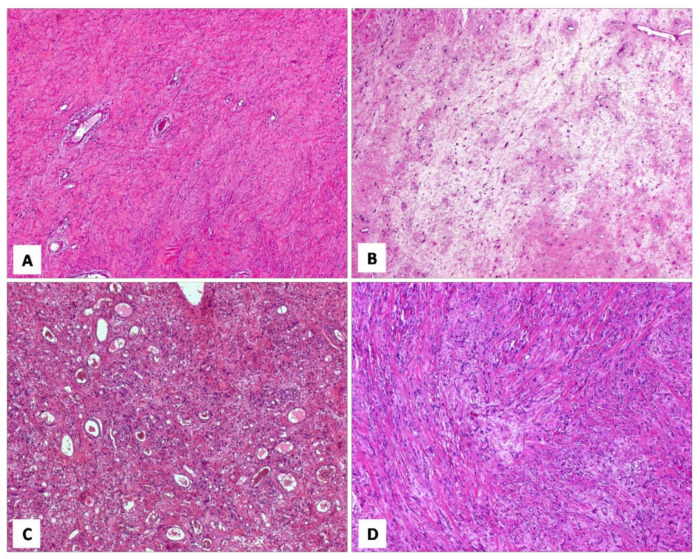
Different histotypes with overlapping morphological features. (**A**) DAM: area with diffuse, collagenized stroma, mimicking CAF (H and E, original magnification 50×). (**B**) CAF: area with hypocellular myxoedematous stroma, mimicking DAM (H and E, original magnification 50×). (**C**) AMFB: area composed exclusively of spindled cells, mimicking CAF (H and E, original magnification 50×). (**D**) MFB: area with linear arrangement of neoplastic cells, mimicking AMFB (H and E, original magnification 200×).

Differential diagnosis of the vulvovaginal stromal tumours may be challenging, as they share several clinical, morphological (Figure 11), immunohistochemical and genetic features. A correct nosological classification is not a mere academic exercise but crucial to differentiate tumours with benign biological behaviour (MFB, AMFB) from locally aggressive tumours (DAM) and from tumours with a low risk of malignant transformation (CAF). The most salient diagnostic features and the differential diagnostic clues are provided in the comparative Table 9, Table 10 and Table 11. CAF and MFB share a cellular composition (bland-looking spindle cells), the oedematous-to-fibrous stroma, the loss of nuclear RB1 expression by immunohistochemistry and the deletion of the 13q14 region by F.I.S.H. analyses. Unlike CAF—which is a subcutaneous tumour—MFB is a subepithelial-centred lesion and, as the name implies, it exhibits a more prominent vascular component than does MFB. In most cases, CAF shows a fibroblastic (CD34+/desmin-) rather than a myofibroblastic profile (desmin+). Occasionally, both CAF and MFB with extensive oedematous stroma may mimic DAM; however, the latter tumour is deep-seated, uniformly hypocellular and has infiltrative margins. Unlike DAM, which is usually a desmin-positive tumour with retained nuclear expression of RB1, CAF is desmin-negative, with the loss of nuclear RB1 immunoreactivity. Although DAM and MFB are desmin-positive tumours, these tumours harbour a different molecular signature; the former is often HMGA2-positive, as a surrogate of *HMGA2* rearrangements, while the latter shows the absence of RB1 nuclear expression and the overexpression of CY2E1, respectively, as a surrogate of the 13q14 deletion and *MTG1–CYP2E1* fusion transcripts [5]. AMFB may also be difficult to distinguish from DAM, CAF and MFB. AMFB differs from DAM in that the former has sharply circumscribed margins and epithelioid cells clustered around capillary-sized vessels. Conversely, DAM has infiltrative margins and contains larger and thicker-walled vessels. AMFB can be distinguished from CAF in that the latter has larger vessels with thick hyalinized walls, in contrast to the thin-walled, capillary-like vessels seen in AMFB. Finally, unlike MFB, AMFB is subcutaneously located and, at least focally, contains epithelioid cells with a perivascular arrangement. In contrast to CAF, AMFB does not show monoallelic deletions of *RB1* and *FOXO1* at the 13q14 locus but *MTG1–CYP2E1* fusion transcripts are commonly identified.

## 5. Conclusions

Based on our experience in approaching the diagnosis of the benign stromal tumours of the lower female genital tract, namely DAM, CAF, AMFB and MFB, we strongly suggest adopting the following recommendations: (i) the diagnosis should be mainly based on histological features in combination with clinical and macroscopic features; (ii) immunohistochemical analyses may be misleading for correct tumour classification due to the non-specific results in the different histotypes; however, a diffuse desmin immunoreactivity in a tumour with abundant myxoid stroma is highly suggestive of DAM; (iii) in cases with ambiguous morphological and immunohistochemical features, F.I.S.H. analysis showing a 13q14 deletion is helpful in ruling out the diagnosis of DAM and AMFB; similarly, the detection of *HMGA2* rearrangements by means of immunohistochemistry or molecular biology is helpful for the diagnosis of DAM; (iv) if the pathologist is dealing with a tumour exhibiting overlapping morphological features among the different histotypes (especially for CAF, AMFB and MFB) it could be a trivial problem to try to subtype a specific tumour at any cost and the use of the generic term “benign stromal tumour of the lower female genital tract” seems to be appropriate.

## Figures and Tables

**Table 1:** Key diagnostic features of DAM.

**Definition**
▪ locally infiltrative, non-metastasizing mesenchymal tumour that occurs in the deep, soft tissues of the vulvovaginal and pelviperineal regions
**Clinical Features**
▪ reproductive-aged females (20–50 years); peak incidence in the third decade ▪ slowly growing, large-sized mass with finger-like projections infiltrating the surrounding soft tissues ▪ often confused with a Bartholin gland cyst or inguinal hernia
**Gross Pathology**
▪ relatively circumscribed, lobulated or polypoid, myxoid or fibro-myxoid mass ▪ tumour size ranges from a few centimetres to 20 cm or more
**Histopathology**
▪ uniformly hypocellular tumour ▪ infiltrative margins with entrapment of adipose tissue, skeletal muscle and nerve fibres ▪ bland-looking, small-sized spindled cells with delicate bipolar cytoplasmic processes and a minority of stellate-shaped cells, haphazardly interspersed in an abundant myxoedematous stroma with wispy collagen fibres ▪ numerous small-, medium- or large-sized blood vessels ▪ mitoses are absent or rare
**Immunohistochemistry/Molecular Diagnostic Features**
▪ diffuse expression of desmin; variable expression of HMGA2 (60–70% of cases) ▪ F.I.S.H.: rearrangements of the HMGA2 locus are identified in approximately 1/3 of tumours
**Treatment/Prognosis**
▪ local recurrence, especially if the tumour is incompletely excised ▪ wide excision with clear margins of at least 1 cm is the preferred treatment. ▪ given its infiltrative nature, complete resection cannot be frequently achieved ▪ DAM may be responsive to gonadotropin-releasing hormone (GnRH) agonists

**Table 2:** Unusual morphological features of DAM [13].

▪ alternating myxoid or fibrous areas ▪ perivascular hyalinization (Figure 2B) ▪ fibrotic obliteration of the vascular lumens (Figure 2C) ▪ fibrous areas with neurofibroma-like appearance (Figure 3B) ▪ hypercellularity, often with perivascular arrangement (Figure 3C) ▪ microcystic/reticular stromal changes ▪ perivascular cuffing with onion-skin arrangement ▪ capillary-like blood vessels with a microvascular growth pattern (as seen in glioblastoma) (Figure 2D) ▪ nodular leiomyomatous differentiation ▪ fibrosclerotic stroma in both primary and recurrent tumours (Figure 3D)

**Table 3:** Key diagnostic features of CAF.

**Definition**
▪ benign stromal tumour, usually occurring in the subcutaneous tissue of the vulvovaginal region of middle-aged women
**Clinical features**
▪ superficially located, slowly growing and painless mass, ranging in size from 0.6 to 25 cm, frequently resembling a Bartholin gland cyst
**Gross pathology**
▪ round or lobulated tumour ▪ well-circumscribed margins ▪ soft-to-rubbery consistency ▪ greyish-to-whitish cut surface
**Histopathology**
▪ well-circumscribed, unencapsulated tumour with entrapped adipose tissue at the periphery ▪ moderately cellular lesion ▪ proliferation of bland-looking spindled cells with the appearance of fibroblasts ▪ prominent fibrous stroma containing wispy collagen bundles ▪ numerous small- to medium-sized blood vessels, often with thick, hyalinized walls ▪ neoplastic cells are haphazardly distributed throughout the tumour ▪ fascicular arrangement or nuclear palisading are often seen ▪ mitotic figures are absent to rare
**Immunohistochemistry/Molecular Diagnostic Features**
▪ CD34, ER and PR are frequently expressed; in 10–20% of cases, myogenic markers (α-smooth-muscle actin; desmin, h-caldesmon) can be variably expressed; loss of nuclear staining for RB1 is common. ▪ F.I.S.H.: monoallelic deletions of *RB1* and/or *FOXO1* at the 13q14 locus
**Treatment/Prognosis**
▪ benign tumour ▪ rare local recurrence ▪ recommended treatment is conservative local excision

**Table 4:** Unusual morphological features of CAF.

▪ dermal location [19] ▪ minimally infiltrative margins [17,19,21] ▪ focal myxoid areas (Figure 5B) ▪ mild cytological atypia [19] ▪ high mitotic activity (up to 11 mitoses/10HPF can be occasionally seen) [16,19] ▪ necrosis [19] ▪ dilated blood vessels with a hemangiopericytoma-like pattern [20,21] (Figure 5A) ▪ edematous/chronic inflammation of blood vessels with thrombotic obliteration of their lumen [19,20,21] ▪ stromal, sometimes perivascular, lymphoid aggregates [20] ▪ pseudovascular spaces filled with proteinaceous fluid [21] ▪ pseudoangiomatous stromal changes, as seen in spindle-cell lipomas [26] ▪ microcystic stromal changes [20] ▪ solitary, fibrous, tumour-like areas [19] ▪ nuclear atypia (so-called “*atypical cellular angiofibroma*”) [21,22,23,24] ▪ sarcomatous transformation/overgrowth in the form of well-differentiated liposarcoma, pleomorphic liposarcoma, spindle-cell sarcoma not otherwise specified or undifferentiated pleomorphic sarcoma [22,23,24]

**Table 5:** Key diagnostic features of AMFB.

**Definition**
▪ benign stromal tumour that mainly involves subcutaneous tissue of the vulva and vagina of women in the reproductive years
**Clinical features**
▪ painless, slowly growing superficially located mass, measuring less than 5 cm in maximum diameter, frequently misinterpreted as a Bartholin gland cyst
**Gross pathology**
▪ unencapsulated or partially/totally encapsulated tumour with well-circumscribed margins ▪ soft-to-rubbery consistency ▪ greyish-pink-to-yellowish-brown cut surface
**Histopathology**
▪ unencapsulated and well circumscribed ▪ alternating hypercellular and hypocellular areas ▪ bland-looking oval, spindled-to-epithelioid/plasmacytoid cells ▪ cells arranged singly or in small nests or cords ▪ bi- or multi-nucleated neoplastic cells ▪ perivascular clustering of neoplastic cells ▪ small-sized, thin-walled, capillary-like blood vessels ▪ mitotic figures are rare or absent ▪ myxoedematous-to-fibrous collagenous stroma ▪ scattered stromal mast cells and lymphocytes ▪ a minority of tumours (10% of cases) may contain islands of mature adipose tissue
**Immunohistochemistry/Molecular Features**
▪ expression of desmin and α-smooth-muscle actin (40% of cases); common expression of CYP2E1, ER, PR, AR, Bcl-2 and CD99 ▪ molecular analyses: MTG1–CYP2E1 fusion transcripts ▪ F.I.S.H.: lack of monoallelic deletions of *RB1* and *FOXO1* at the 13q14 locus
**Treatment/Prognosis**
▪ benign tumour ▪ conservative local excision with clear margins ▪ rare local recurrence

**Table 6:** Unusual morphological features of angiomyofibroblastoma.

▪ diffuse hypercellularity [36] ▪ diffuse hypocellularity [36,42] ▪ minimal nuclear atypia [27,34] ▪ mitotic activity: up to 7 mitoses/50 HPF tumour (“mitotically active AMFB”) [43,44] ▪ prominent cord-like growth pattern reminiscent of a Sertoli cell tumour, sclerosing type [36] ▪ short fascicular, wavy or palisading cellular proliferation [45] ▪ perivascular fibrosclerosis [5] ▪ blood-filled, pseudo-angiomatoid, cystic spaces resembling an angiomatoid fibrous histiocytoma [46] ▪ capillary haemangioma-like appearance [45] ▪ overlapping/intermediate morphological features with MFB [10], CAF [47] and DAM [10,47] ▪ prominent fatty component (lipomatous angiomyofibroblastoma) [35,37] (Figure 7A) ▪ atypical cells and high mitotic activity [40] ▪ sarcomatous areas resembling spindle-cell pleomorphic sarcoma [41]

**Table 7:** Key diagnostic features of MFB.

**Definition**
▪ benign myofibroblastic spindle-cell tumour arising from the subepithelial stroma of the vagina and, less frequently, of the vulva or cervix.
**Clinical Features**
▪ age (23–80 years). ▪ a subset of patients have a history of hormonal or tamoxifen therapy ▪ slowly growing, painless mass
**Gross Pathology**
▪ soft-to-firm in consistency ▪ whitish in colour ▪ well-circumscribed, occasionally lobulated margins
**Histopathology**
▪ well-circumscribed margins; rarely focally infiltrative ▪ variable cellularity ▪ **superficial-type**: spindled/stellate cells set in a loose, oedematous, finely collagenous stroma with reticular, lace-like or sieve-like pattern; mild nuclear atypia ▪ **Mammary-type**: spindle cells arranged in short, intersecting fascicles interrupted by keloid-like collagen fibres ▪ low mitotic count (<1 mitosis/10 HPFs) ▪ small-to-medium-sized, often with hyalinized walls ▪ mast cells variably interspersed among spindle cells
**Immunohistochemistry/Molecular Diagnostic Features**
▪ diffuse expression of desmin; variable expression of α-smooth-muscle actin, CYP2E1, CD34, oestrogen/progesterone/androgen receptors, CD10, bcl-2, CD99; loss of nuclear RB1 expression is common ▪ F.I.S.H.: monoallelic loss of *RB1* and *FOXO1* at the 13q14 locus (90% of cases) ▪ molecular analyses: *MTG1–CYP2E1* fusion transcripts
**Treatment/Prognosis**
▪ complete surgical excision ▪ clinical indolent course without local recurrence

**Table 8:** Unusual morphological features of MFB.

▪ **superfical-type**: - absence of the Grenz zone [50,52] - multinucleated cells [50,52] - focal areas composed of small, round, blue cells [52] (Figure 10A,B) - mild cytological atypia [50,51] ▪ **mammary-type**: - diffuse, fibrosclerotic stroma (so-called “collagenized/fibrotic MFB”) [52] (Figure 10C) - single-cell file, cord-like growth patterns [52] (Figure 10D) - neurofibroma-like or focal storiform growth patterns [52]

**Table 9:** Clinicopathologic features of the stromal tumours of the lower female genital tract.

Tumor	DAM	CAF	AMFB	MFB
**localization**	deep soft tissues	dermal/subcutaneous	subcutaneous	sub-epithelial/dermal lesion; the surgical samples often contain overlying vaginal mucosa or vulvar skin
**hmargins**	minimally/widely infiltrative; entrapment of adipose tissue and/or skeletal muscle and/or nerve fibres	circumscribed; occasionally minimally infiltrative	circumscribed	well circumscribed; band of native connective tissue (Grenz zone) between tumour and overlying epithelium
**cellularity**	uniformly hypocellular	uniformly cellular	alternating hypercellular and hypocellular areas	variable
**cytology**	small-sized, spindled-to-stellate cells	spindled cells, often with bipolar processes and wavy appearance (perineuroma-like resemblance)	spindled/epithelioid cells	spindled/stellate cells
**mitoses**	rare	rare; occasionally up to 3 mitoses/10HPF	rare	rare
**growth pattern**	haphazard arrangement	haphazard arrangement; short fascicles	perivascular arrangement; single cells, nests, cords,	single cells; reticular, lace-like or sieve-like pattern; short fascicles.
**vasculature**	numerous small-to-medium-to-large-sized blood vessels	numerous small-to-medium-to-large-sized blood vessels, often with hyalinized walls	numerous small-sized, thin-walled, capillary-like vessels	small-to-medium-sized blood vessels, often with hyalinized walls
**stroma**	uniformly myxoedematous with wispy collagen fibres	fibrous to focally myxoid with wispy collagen fibres	myxoid-to-focally fibrous	oedematous-to-finely-collagenous stroma; isolated thick collagen bands may be seen
**metaplastic component**	small bundles of mature, smooth-muscle cells (30% of cases)	mature fatty tissue (30% of cases)	mature, fatty tissue (10% of cases)	not reported
**sarcomatous transformation**	not reported	rare: single or multiple nodules of pleomorphic sarcoma	very rare: spindle-cell sarcoma	not reported
**immunophenotype**	desmin-positive; variable expression of α-smooth-muscle actin and HMGA2	CD34-positive; loss of nuclear RB1 expression; variable expression of α-smooth-muscle actin; desmin is usually negative	CYP2E1-positive; variable expression of desmin (40–50% of cases), α-smooth-muscle actin and CD34;	desmin-positive; variable expression of CD34 and α-smooth-muscle actin; loss of nuclear RB1 expression
**molecular analyses**	*HMGA2* rearrangements	monoallelic loss of *RB1* and *FOXO1* at the 13q14 locus (F.I.S.H.)	*MTG1–CYP2E1* fusion transcripts	monoallelic loss of *RB1* and *FOXO1* at the 13q14 locus (F.I.S.H.); *MTG1–CYP2E1* fusion transcripts

**Table 10:** Differential diagnosis: DAM vs. the other stromal tumours.

DAM vs. CAF
**Shared Features**: ▪ bland-looking spindled cells ▪ prominent vascular component
**Distinguishing Features**: ▪ **site:** DAM involves deep, soft tissues; CAF is typically a subcutaneous lesion ▪ **tumour margins:** DAM has, at least focally, infiltrative margins ▪ **cellularity:** DAM is a more hypocellular tumour than CAF ▪ **cellular composition:** DAM has small-sized spindled-to-stellate cells; CAF has more elongated spindled cells (fibroblastic-like appearance) ▪ **tumour stroma:** DAM is more myxoid than CAF ▪ **vascular component:** DAM has larger blood vessels than CAF; the blood vessels of CAF often show hyalinization of their walls ▪ **immunohistochemistry:** DAM is commonly desmin+/CD34-, whereas CAF is usually CD34+/desmin-; CAF often shows the loss of nuclear RB1 expression that is maintained in DAM ▪ **molecular analyses:** CAF shows monoallelic loss of *RB1* and *FOXO1* at the 13q14 locus; DAM may show *HMGA2* rearrangements
**DAM vs. MFB**
**Shared Features**: ▪ bland-looking spindle cells ▪ desmin immunoreactivity
**Distinguishing Features**: ▪ **localization:** DAM is a more deep-seated tumour; MFB is subepithelial-centred ▪ **margins:** DAM has, at least focally, infiltrative margins ▪ **cellularity:** DAM is a more uniformly hypocellular tumour ▪ **tumour stroma:** DAM is a more uniformly myxoid tumour ▪ **vascular component:** DAM has a more prominent vascularization consisting of larger blood vessels than are seen in MFB ▪ **immunohistochemistry:** MFB is CYP2E1-positive and shows a loss of nuclear RB1 expression ▪ **molecular analyses:** MFB shows monoallelic deletions of *RB1* and *FOXO1* at the 13q14 locus (F.I.S.H.) and *MTG1–CYP2E1* fusion transcripts; DAM may show *HMGA2* rearrangements
**DAM vs. AMFB**
**Shared Features**: ▪ bland-looking spindled cells ▪ prominent vascular component ▪ desmin immunoreactivity
**Distinguishing Features**: ▪ **localization:** DAM is deep-seated; AMFB is a subcutaneous-centred tumour ▪ **margins:** DAM has, at least focally, infiltrative margins ▪ **cellularity:** DAM is a more uniformly hypocellular tumour ▪ **cellular composition:** AMFB has often epithelioid cells ▪ **growth pattern:** AMFB exhibits perivascular clustering of neoplastic cells ▪ **tumour stroma:** DAM is uniformly more myxoid than AMFB ▪ **vascular component:** DAM has blood vessels larger than are seen in AMFB ▪ **immunohistochemistry:** AMFB is CYP2E1-positive ▪ **molecular analyses:** DAM may show *HMGA2* rearrangements; AMFB shows *MTG1–CYP2E1* fusion transcripts

**Table 11:** Differential diagnosis among CAF, AMFB and MFB.

CAF vs. AMFB
**Shared Features** ▪ subcutaneous localization ▪ circumscribed margins ▪ bland-looking spindle cells ▪ prominent vascular component ▪ fibro-myxoid stroma
**Distinguishing Features**: ▪ **cellularity:** CAF is a uniformly cellular tumour, whereas AMFB shows alternating hypercellular and hypocellular areas ▪ **cellular composition:** CAF is a more uniformly spindled tumour; AMFB often contains epithelioid cells ▪ **growth pattern:** CAF has a short fascicular arrangement; AMFB exhibits perivascular clustering of neoplastic cells ▪ **vascular component:** CAF has small-to-medium-sized blood vessels, often with perivascular hyalinization; unlikely, AMFB has thin-walled, capillary-like vessels ▪ **tumour stroma:** CAF has a more fibrous stroma ▪ **immunohistochemistry:** AMFB is commonly desmin- and CYP2E1-positive; unlikely, CAF is more commonly CD34-positive and shows the loss of nuclear RB1 expression ▪ **molecular analyses:** CAF shows monoallelic deletions of *RB1* and *FOXO1* at the 13q14 locus (F.I.S.H.); AMFB shows *MTG1–CYP2E1* fusion transcripts
**CAF vs. MFB**
**Shared Features**: ▪ circumscribed margins ▪ bland-looking spindle cells ▪ blood vessels with hyalinized walls ▪ CD34 expression ▪ loss of nuclear RB1 expression by immunohistochemistry ▪ monoallelic deletions of the 13q14 region (*RB1* and *FOXO1*)
**Distinguishing Features**: ▪ **localization:** MFB is a sub-epithelial-centred tumour ▪ **cellularity:** CAF is uniformly more cellular than MFB ▪ **tumour stroma:** CAF has a more fibrous stroma ▪ **vascular component:** CAF has a more prominent vascularization ▪ **immunohistochemistry:** unlike CAF, MFB is typically desmin-positive ▪ **molecular analyses:** MFB shows *MTG1–CYP2E1* fusion transcripts
**AMFB vs. MFB**
**Shared Features**: ▪ circumscribed margins ▪ bland-looking spindle cells ▪ fibromyxoid stroma ▪ desmin immunoreactivity ▪ CYP2E1 immunoreactivity ▪ *MTG1–CYP2E1* fusion transcripts
**Distinguishing Features**: ▪ **localization** MFB is a sub-epithelial-centred tumour ▪ **cellular composition:** AMFB often has epithelioid cells ▪ **growth pattern:** AMFB shows perivascular clustering of neoplastic cells ▪ **immunohistochemistry:** MFB is more diffusely and strongly positive to desmin and often shows a loss of nuclear RB1 expression ▪ **molecular analyses:** MFB often shows monoallelic deletions of *RB1* and *FOXO1* at the 13q14 locus (F.I.S.H.)

## Data Availability

All data presented in this manuscript are available from the corresponding author upon reasonable request.
